# Carbohydrate Conjugates in Vaccine Developments

**DOI:** 10.3389/fchem.2020.00284

**Published:** 2020-04-15

**Authors:** Shuyao Lang, Xuefei Huang

**Affiliations:** ^1^Department of Chemistry, Michigan State University, East Lansing, MI, United States; ^2^Institute for Quantitative Health Science and Engineering, Michigan State University, East Lansing, MI, United States; ^3^Department of Biomedical Engineering, Michigan State University, East Lansing, MI, United States

**Keywords:** adjuvant, carbohydrates, immune activation, vaccine development, glyco-conjugates

## Abstract

Vaccines are powerful tools that can activate the immune system for protection against various diseases. As carbohydrates can play important roles in immune recognition, they have been widely applied in vaccine development. Carbohydrate antigens have been investigated in vaccines against various pathogenic microbes and cancer. Polysaccharides such as dextran and β-glucan can serve as smart vaccine carriers for efficient antigen delivery to immune cells. Some glycolipids, such as galactosylceramide and monophosphoryl lipid A, are strong immune stimulators, which have been studied as vaccine adjuvants. In this review, we focus on the current advances in applying carbohydrates as vaccine delivery carriers and adjuvants. We will discuss the examples that involve chemical modifications of the carbohydrates for effective antigen delivery, as well as covalent antigen-carbohydrate conjugates for enhanced immune responses.

## Introduction

Carbohydrates are common surface molecules in the living system. With their rich structural diversities, carbohydrate molecules play important roles in cellular recognition and signaling, including immune recognition, and activation (Rabinovich et al., 2012; Mahla et al., 2013; Varki, 2016). Most of the cell surface immune receptors, such as toll-like receptors (TLRs), NOD-like receptors (NLRs) and major histocompatibility complex class I and class II (MHC I and MHC II), are glycoproteins. Several essential receptors for immune cell activation, e.g., TLRs, NLRs, C-type lectins, and sialic acid-binding immunoglobulin-type lectins (Siglecs), can recognize glycan containing ligands including those expressed on the surface of many pathogenic microbes and cancer cells (Rabinovich et al., 2012).

Carbohydrates have been widely applied in vaccine development (Lesinski and Westerink, 2001). Vaccines containing bacterial polysaccharides have been commercialized as anti-bacterial vaccines (Roy, 2004; Astronomo and Burton, 2010), and many anti-cancer vaccines have been studied to target tumor-associated carbohydrate antigens (TACAs) (Guo and Wang, 2009; Astronomo and Burton, 2010; Yin and Huang, 2012; Feng et al., 2016). Carbohydrates are also attractive immune adjuvant candidates. Various carbohydrates such as β-glucan, mannan, and monophosphoryl lipid A (MPLA) can activate the immune system and induce T helper cell type 1 (Th1) immune responses (Suzuki et al., 2001; Stambas et al., 2002; Petrovsky and Cooper, 2011; Hu et al., 2013). They may complement Alum, the FDA approved adjuvant in humans, which only induces T helper cell type 2 (Th2) immune responses. Carbohydrates can be readily metabolized or degraded *in vivo* and are less likely to generate long-term toxicity (Petrovsky and Cooper, 2011; Hu et al., 2015; Li and Wang, 2015). With their biocompatibility, low toxicity and ease of modification, carbohydrates have been studied as carriers for antigen delivery (Liu et al., 2008; Correia-Pinto et al., 2013; Zhang et al., 2013; Cordeiro et al., 2015; Pushpamalar et al., 2016), which can often induce immune cell targeting and provide self-adjuvanting activities for a successful vaccination.

Although natural carbohydrates can be applied as vaccine components directly (Mata-Haro et al., 2007; Arca et al., 2009; Mirza et al., 2017). in many cases chemical modification of carbohydrates is necessary for enhanced efficacy. One of the commonly used strategies in vaccine design is to prepare conjugates of antigens and/or adjuvants with the delivery carrier (Liu and Irvine, 2015). This can be beneficial in multiple ways, such as prolonged circulation and controlled release, size-induced lymph node targeting, better immune recognition through multivalency, enhanced cell uptake and immune activation. In this review, we focus on recent vaccine designs applying carbohydrates as vaccine delivery carriers and adjuvants. We will discuss examples involving chemical modifications of the carbohydrates, especially the covalent conjugates of antigens and carbohydrate-based delivery carrier or adjuvants. Vaccines that contain carbohydrates and derivatives only as antigen components, or natural carbohydrates encapsulated/admixed with other vaccine components, have been reviewed (Marzabadi and Franck, 2017; Colombo et al., 2018; Wei et al., 2018; Weyant et al., 2018; Jin et al., 2019; Micoli et al., 2019), and are not discussed here.

## Zwitterionic Polysaccharides (ZPSs)

**Scheme 1:**
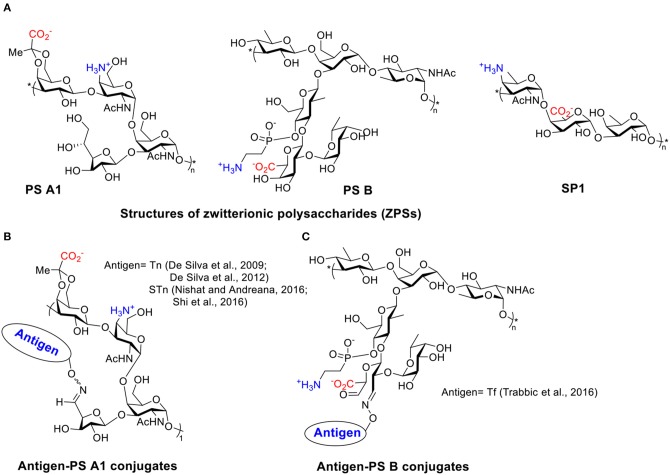
**(A)** Structures of several ZPSs. **(B)** Examples of antigen-PS A1 conjugates. **(C)** Examples of antigen-PS B conjugates.

Many types of bacteria can produce high molecular weight polysaccharides as their capsules. Polysaccharides have been traditionally considered as T cell independent antigens unless conjugated to proteins or lipids (Stein, 1992; Wei et al., 2018). Polysaccharides usually interact with polysaccharide-specific B cells generating low-affinity IgM with little detectable IgG antibodies and little induction of T cell responses or immune memory (Abbas et al., 2000). However, a special group of polysaccharides, referred to as ZPSs, has been found to have the ability to induce MHC II mediated T cell response specifically (Kalka-Moll et al., 2002; Mazmanian and Kasper, 2006). At least eight different ZPSs have been isolated from *Bacteroides fragilis, Staphylococcus aureus*, and *Streptococcus pneumoniae type 1*, of which the PS A1 (isolated from *Bacteroides fragilis*) is the most studied ZPS so far (Scheme 1A) (Cobb and Kasper, 2005; Mazmanian and Kasper, 2006; Surana and Kasper, 2012; Nishat and Andreana, 2016).

TACAs are saccharides aberrantly expressed on surfaces of multiple types of cancer cells (Heimburg-Molinaro et al., 2011). Like most types of carbohydrate antigens, TACAs induce only weak IgM responses when administered alone. For successful TACA vaccines, TACAs are commonly conjugated with strong immunogenic proteins, such as bovine serum albumin (BSA), tetanus toxoid (TT), keyhole limpet hemocyanin (KLH), and virus like particles, in order to generate high levels of IgG responses (Kaltgrad et al., 2007; Heimburg-Molinaro et al., 2011; Wu et al., 2018, 2019). However, these carrier proteins can result in carrier induced suppression of antibody responses to the desired TACA due to high antibody responses to the carrier itself (Leclerc et al., 1990). Furthermore, some of the protein carriers tend to aggregate or suffer from stability issues (Dasgupta et al., 2014). ZPSs as novel non-protein T cell-activating carriers have been applied to cancer vaccine design by the Andreana group (De Silva et al., 2009). They first reported an “entirely carbohydrate vaccine” by conjugating a model TACA, Tn, and the most studied type of ZPS, PS A1. PS A1 was isolated from *B. Fragilis* in a large scale, then subjected to selective oxidation leading to aldehyde functioned PS A1 that reacted with aminooxy functionalized Tn by oxime formation (Scheme 1B).

Immunization of mice with Tn-PS A1 resulted in a 200-fold increase of total antibody titer against Tn compared to the pre-immunized sera, while the antibody titers against the PS A1 backbone were modest. IgM and IgG3 were the major subtypes of antibodies generated (De Silva et al., 2009). Anti-sera of Tn-PS A1 immunized mice were found to react with a range of Tn expressing cancer cell lines (MCF-7, MDA-231, Jurkat, JurkatTAg, Panc-1) (De Silva et al., 2012), while binding little to human peripheral blood mononuclear cells and human bone marrow cells as the negative control. The anti-PS A1 and anti-Tn-PS A1 sera showed completely different cytokine profiles. A high level of IL-17A, a pro-inflammatory factor promoting CD4^+^ T cell proliferation, was detected in anti-Tn-PS A1 sera but not in anti-PS A1 sera. Besides Tn antigen, other TACAs such as sialyl-Tn (STn) (Nishat and Andreana, 2016; Shi et al., 2016) and Thomsen-Friedenreich (Tf) (Trabbic et al., 2016) have been conjugated with PS A1 (Scheme 1B) and another ZPS, i.e., PS B (Scheme 1C) (Trabbic et al., 2016). The conjugates were able to induce moderate levels of both IgM and IgG antibodies against the target TACAs. Co-administration of an exogenous adjuvant such as Sigma adjuvant system (SAS) and TiterMax Gold (TMG) could enhance the levels of IgG antibodies. Post-immune sera bound with multiple types of cancer cells and were able to kill tumor cells via complement-dependent cytotoxicities while sparing normal cells. Furthermore, the STn-PS A1+SAS vaccine generated cellular immunity besides humoral antibody response. The enzyme-linked immune absorbent spot (ELISpot) assay of splenocytes from mice immunized with STn-PS A1+SAS pulsed with STn-PS A1 or BSM showed secretion of INF-γ, clearly indicating a Th1-dominant cellular immune response.

These studies indicated that ZPSs are promising vaccine carrier/adjuvant to elicit a selective immune response against TACAs. However, to date, the efficacy of protection in mouse tumor models by these entirely carbohydrate vaccines have not been reported. Further studies are needed to demonstrate the full potential of ZPS in anti-cancer vaccine development.

## MPLA

**Scheme 2:**
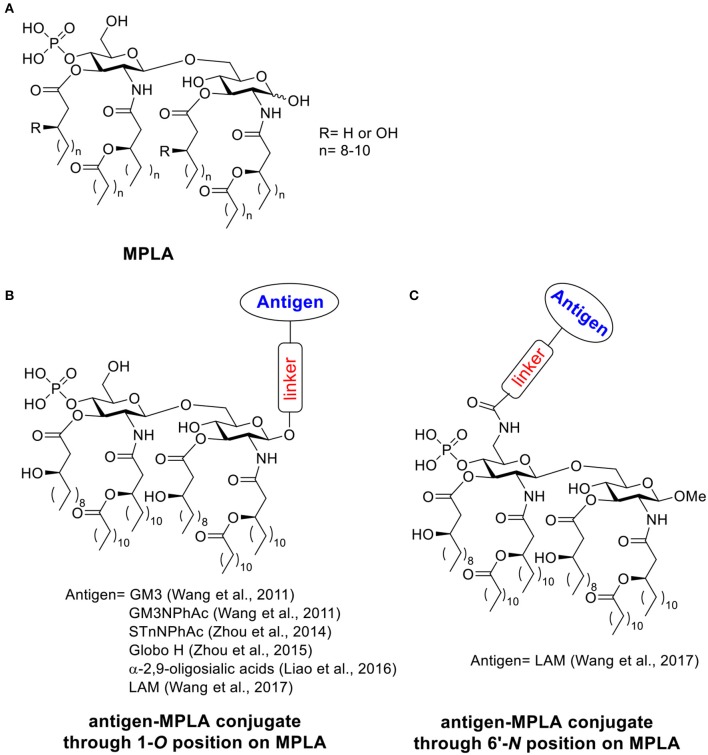
**(A)** Structure of MPLA. **(B)** Examples of antigen-MPLA conjugate through 1-*O* position. **(C)** Example of antigen-MPLA conjugate through 6′-*N* position on MPLA.

MPLA is a derivative of lipopolysaccharide (LPS), a fraction isolated from cell walls of gram-negative bacteria such as *Salmonella minnesota* (Casella and Mitchell, 2008). Through a hydrolytic process reported by Edgar Ribi, LPS can be converted into an acylated di-glucosamine mixture widely known as monophosphoryl lipid (Ribi et al., 1979; Qureshi et al., 1982; Casella and Mitchell, 2008). The majority of these species contains six acyl side chains, no polysaccharide chains and one phosphoryl group (Scheme 2A) (Evans et al., 2003; Casella and Mitchell, 2008). Compared to LPS, MPLA is about 0.1% as toxic as the parent LPS compound in rabbit pyrogenicity assays while maintaining its immune-stimulating activities (Qureshi et al., 1982; Evans et al., 2003). MPLA interacts with the immune system through TLR-4 and usually induces Th1 or a blended Th1 and Th2 type immune response. With its low toxicity, MPLA has been applied as the adjuvant in several vaccines successfully in clinical trials (Evans et al., 2003; Cluff, 2009; Artiaga et al., 2016). Vaccines containing MPLA such as FENDrix (HBV vaccine), Cervarix (HPV vaccine), Melacine (melanoma vaccine), Pollinex Quattro (allergy vaccine), and Mosquirix (malaria vaccine for young children) have been registered for use in many countries (Artiaga et al., 2016). MPLA can also serve as a vaccine carrier and a built-in adjuvant when conjugated with antigens covalently. Herein we discuss examples of fully synthetic vaccines containing MPLA as the carrier (Wu and Guo, 2006; Wang et al., 2011; Zhou et al., 2014, 2015; Liao et al., 2016).

In 2011, the Guo lab first reported the covalent conjugation of a TACA, i.e., GM3, with MPLA as an anti-cancer vaccine (Wang et al., 2011). The liposomal vaccine was formed by sonication of a mixture of the GM3-MPLA conjugate, 1,2-distearoyl-sn-glycero-3-phosphocholine, and cholesterol. The resulting vaccine was injected to C57BL/6 mice subcutaneously for 4 weekly injections. A strong GM3-specific antibody response was observed by enzyme-linked immuno-sorbent assay (ELISA) in antisera on day 38, which included high levels of both IgM and IgG3 antibodies. When a GM3 derivative, GM3NPhAc (Pan et al., 2005), was conjugated with MPLA using a similar strategy, a 3.8 times higher total antibody titer with a significant increase of IgG3 and IgG1 titers was observed in day 38 antisera compared to the GM3-MPLA group. The antisera from GM3NPhAc-MPLA immunized mice showed strong binding toward cancer cell SKMEL-28 by fluorescence activated cell sorting (FACS) analysis. The free phosphate and free hydroxyl groups on MPLA are important for immunostimulation, as the conjugates with benzyl protected phosphate and hydroxyl groups showed no significant immune responses. The linker between MPLA and GM3/GM3NPhAc did not significantly influence the immunological properties of the resulting conjugates. Interestingly, addition of an external adjuvant such as Titermax Gold to the vaccine formulation led to lower antibody titers relative to GM3/GM3NPhAc-MPLA conjugates alone. This work indicated that the fully synthetic conjugation of MPLA-TACA can serve as a possible “self-adjuvanting” cancer vaccine candidate.

The generality of the MPLA platform has been demonstrated in later studies. Three more MPLA analogs with different lipid chain lengths and linkages were synthesized and conjugated to another TACA derivative, STnNPhAc (Wu and Guo, 2006; Zhou et al., 2014), and formulated into a liposomal vaccine. All STnNPhAc-MPLA conjugates successfully generated immune responses toward STnNPhAc in mice and the conjugate with an 8-carbon lipid chain length and free -OH groups induced the highest antibody titers. Similar to the GM3-MPLA conjugate, when the exogenous adjuvant Titermax Gold was added to the formulation, the antibody titers decreased.

The optimized MPLA structure was used to conjugate with another TACA, globo H, and the immunological properties were compared with the globo H conjugate with KLH, a gold standard carrier commonly utilized in vaccine studies (Zhou et al., 2015). Significantly higher total antibody titers as well as IgG titers were observed in anti-sera from MPLA-globo H immunized mice compared to those immunized with KLH-globo H, suggesting the advantage of MPLA as the carrier. Both conjugates induced higher levels of pro-inflammatory cytokines including IL-4, IL-12, IFN-γ, and TNF-α in mice compared to the non-immunized group. Although the KLH-globo H group showed a higher level of cytokine secretion compared to MPLA-globo H, antisera from MPLA-globo H immunized mice showed a stronger binding toward both MCF-7 and SKMEL-28 tumor cells by FACS analysis and induced more cell lysis of human breast cancer cell MCF-7. The enhanced cytokine secretion in KLH conjugate group might come from the immune response against the protein carrier instead of the globo H antigen. This study indicated that the MPLA may serve as a good alternative to KLH protein vaccine carrier.

In addition to the aforementioned cancer vaccines, a Group C meningitis vaccine has been reported by conjugating MPLA and α-2,9-oligosialic acid containing di-, tri-, tetra- and penta-sialic acid (Liao et al., 2016). The resulting liposomal vaccines with various MPLA-oligosialic acid conjugates induced strong immune responses as revealed by high total antibody titers. The major antibody subtype generated was IgG2b indicating a T cell-dependent immunity. Both oligosialic acid chain length and MPLA structure influenced the immune responses. The shorter sialic acid chains (di- and tri-sialic acid) were overall better immunogens than longer ones (tetra- and penta-sialic acid). However, the antibody induced by the short sialic acid were more restricted to short sialic acid chains. Conjugates containing tri-, tetra-, or penta-sialic acid showed stronger binding toward Group C *meningitides* capsule polysaccharide than the conjugate containing di-sialic acid. Consistent with cancer vaccine studies, addition of external adjuvants such as CFA, alum and Titermax Gold did not lead to higher antibody responses. All conjugates showed protective effects against Group C *meningitides* bacterial challenges in mice, which suggested the possibility of applying the MPLA platform to anti-microbial vaccine development.

In the aforementioned MPLA based vaccine designs, the antigens were all conjugated with MPLA through 1-*O*-position instead of 6′-*O*-position where the polysaccharide chain is attached to LPS in nature (Wang et al., 2017). Guo and Gu further studied the influence of different antigen linkage positions on immunological properties (Schemes 2B,C), by linking a tetrasaccharide antigen from lipoarabinomannan (LAM), a *Mycobacterium tuberculosis* cell surface lipopolysaccharide, to either 1 or 6′ position of MPLA. As the ester linkage on 6′ position was not stable, the 6′-*O* was first substituted with an amino group linker in order to form a more stable amide bond. The resulting conjugates were evaluated *in vivo*. Both conjugates showed significantly enhanced antibody titers against LAM compared to the simple mixture of tetrasaccharide and MPLA, which indicated the importance of covalent conjugation between the antigen and MPLA. As revealed by ELISA, the antigen conjugated to MPLA through 6′-*N* position induced significantly higher IgG titers than the corresponding conjugate through the 1-*O* position. The method of vaccine administration also influenced the immune response outcome. Vaccine given through intraperitoneal injection induced a 4–5 times higher antibody titer compared to the subcutaneous route. This study suggested the conjugation through 6′ position of MPLA could be a more superior strategy for MPLA based vaccine design.

As a low toxicity TLR4 stimulator, MPLA has been widely applied in many vaccines as an add-in adjuvant (Artiaga et al., 2016). Guo's work demonstrated the potential of MPLA as a good “self-adjuvating” vaccine carrier. MPLA-antigen conjugates containing liposomal vaccines can induce strong immune responses comparable to KLH protein. The MPLA platform showed good generality for several carbohydrate antigens including TACAs and bacterial glycans. This platform is not compatible with many external adjuvants and the antigen conjugation site can significantly influence the outcome of vaccination.

## Mannan

**Scheme 3:**
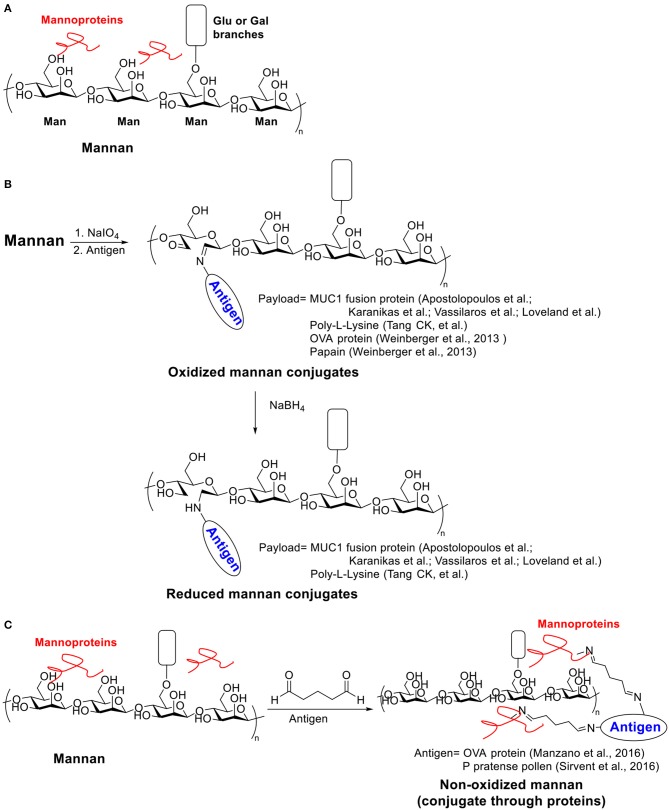
**(A)** Structure of Mannan. **(B)** Examples of oxidized and reduced mannan conjugates. **(C)** Examples of non-oxidized mannan conjugated with antigens through proteins.

Mannan, a polysaccharide derived from the yeast cell wall, contains mostly β-1,4-linked mannose backbone with a small number of α-1,6- linked glucose and galactose side chain residues (Moreira and Filho, 2008). In addition, around 5% proteins were contained in mannan (Scheme 3A) (Nelson et al., 1991; Tzianabos, 2000). As an important component of fungal cell wall, mannan has been widely targeted as carbohydrate based vaccines for Candidiasis (Han and Rhew, 2012; Cassone, 2013; Johnson and Bundle, 2013). It was noticed from patients suffering from Candidiasis that the mannan has immunomodulatory functions (Domer et al., 1986; Wang et al., 1998). Mannan can be recognized through binding with mannose recognition lectins presented on macrophages and other immune cells, which activates the host immune system via a non-self-recognition mechanism (Vasta et al., 1999; Gadjeva et al., 2004). The recognition initiates a set of signal transduction events leading to cytokine secretion, complement activation and CD8^+^ T cell activation (Garner et al., 1990; Garner and Hudson, 1996; Tzianabos, 2000). In this section, we focus on vaccines based on mannan carrier-antigen complex/conjugations, including mannan-mucin 1 (MUC1) fusion protein conjugation for tumor therapy, mannan-DNA vaccine and mannan-allergy vaccines.

The investigation of mannan's potential as a vaccine carrier started in 1990s. The Steward group conjugated mannan and dextran to hepatitis B virus (HBV) 139-147 peptide and studied the immune response in mice toward these two constructs (Okawa et al., 1992). The mannan carrier successfully induced high IgG titers against HBV 139–147 peptide without additional adjuvants, while the corresponding dextran conjugate failed to elicit an immune response. Although some previous studies showed that mannan could suppress immunity (Garner et al., 1990; Podzorski et al., 1990), this study opened the door for using mannan as a “self-adjuvanting” vaccine carrier to enhance antibody production.

### Mannan-MUC1 Fusion Protein Conjugation (M-FP)

Mucins are heavily glycosylated proteins expressed on cell surface. MUC1 is a prototypical mucin, which has been found to be over-expressed on a wide range of tumor cells. Furthermore, tumor associated MUC1 has drastically shorter *O*-glycans in the tandem repeat region of MUC1 made of 20-amino acid residues (APDTRPAPGSTAPPAHGVTS) (Gendler et al., 1990), which leads to the exposure of the protein core, rendering it a highly attractive antigen for anti-cancer immune-therapy (Gendler et al., 1988; Hanisch et al., 1989).

MUC1 by itself is only weakly immunogenic in humans partly due to its self-antigen nature. Immunization of mice with MUC1 fusion protein containing 5 of the tandem repeats induced antibodies but with little measurable cytotoxic T cell (CTL) responses and poor tumor protection (Apostolopoulos et al., 1994). To enhance anti-MUC1 immunity, MUC1 has been conjugated with mannan (Apostolopoulos et al., 1996).

Two strategies (oxidative or reductive, Scheme 3B) for linking mannan to MUC1 have been investigated, which induced drastically different types of immune responses (Apostolopoulos et al., 1995b). Human MUC1 FP was conjugated to mannan oxidized with sodium periodate to provide the oxidative mannan-MUC1 fusion protein conjugate (ox-M-FP). The reductive mannan-MUC1 fusion protein conjugate (red-M-FP) was obtained by treating ox-M-FP with sodium borohydride. BALB/c mice were immunized with either ox- or red- M-FP then challenged with MUC1^+^ 3T3 tumor cells. The red-M-FP generated Th2 type immune responses and induced antibody secretion. However, it has little tumor protective effects. In contrast, the ox-M-FP generated Th1 type responses and induced a high tumor specific CTL precursor frequency providing protection in a mouse tumor model. The CTL response elicited by ox-M-FP was MHC I restricted (Apostolopoulos et al., 1995a), and the CTL precursor frequency can be further enhanced by a combination with a chemotherapeutic drug, i.e., cyclophosphamide (Apostolopoulos et al., 1998). The detailed mechanism of the entry of ox-M-FP into MHC I pathway has also been studied (Apostolopoulos et al., 2000). While both aldehyde and Schiff base groups are presented on ox-M-FP, the aldehyde groups but not the Schiff base groups were found to be important for antigen presentation through the MHC I pathway.

The ox-M-FP had been evaluated in human clinical trials. In phase I studies, no significant toxicities or autoimmunities were noted among >100 patients with advanced melanoma. However, in contrast to preclinical mouse studies, the patients generated mainly antibodies rather than cellular immunity against MUC1 (Karanikas et al., 1997, 2000, 2001). The route of ox-M-FP administration influenced antibody generation in patients. Intraperitoneal injections were significantly more effective compared to intramuscular injections (Karanikas et al., 2001). Pilot phase III study of ox-M-FP has been performed in early-stage breast cancer (Apostolopoulos et al., 2006). Although vaccine-induced antibody and weak cellular immunity responses showed little benefits in advanced disease stage, ox-M-FP significantly improved survival time compared to the placebo control group in early-stage cancer patients (Apostolopoulos et al., 2006). In a 12–15 years follow-up study, the recurrence rate of ox-M-FP group was much lower than that of the placebo group (12.5 vs. 60%) (Vassilaros et al., 2013). The mean time of recurrence in the ox-M-FP group was 52.2 months longer compared to placebo group (118 vs. 65.8 months) (Vassilaros et al., 2013). In another study, autologous dendritic cells were chosen as the vaccine carrier to maximize the cellular immunity in patients (Loveland et al., 2006). The phase I/II clinical trial showed ox-M-FP loaded monocyte derived dendritic cells were well tolerated for immunotherapy, and vaccine-specific IFN-γ secreting CD4^+^ and CD8^+^ T cells were successfully induced in all patients (Loveland et al., 2006).

### Mannan as Carrier for DNA Vaccines

Oxidized and reduced mannan (ox-Man and red-Man, respectively) have been studied as DNA vaccine carriers. Apostolopoulos and Pietersz groups conjugated ox-Man and red-Man with polycationic linker poly-L-lysine (PLL) and then complexed them with DNA corresponding to the protein ovalbumin (OVA) (Tang et al., 2007). The conjugation with mannan reduced cytotoxicity of PLL, and the Man-PLL-OVA DNA complex successfully induced immune responses against OVA. At a lower dose (10 μg), red-Man-PLL-OVA DNA mainly induced CD4^+^ T cell responses, while ox-Man-PLL-OVA DNA induced CD8^+^ T cell responses. Meanwhile, at a higher immunization dose (50 μg), both red-Man and ox-Man-PLL-OVA DNA complex generated CD4^+^ and CD8^+^ T cell responses. Both complexes induced good tumor protection against OVA expressing EG.7 tumor using either low (10 μg) or high (50 μg) immunization doses.

With the success of OVA DNA vaccine, Apostolopoulos and coworkers further studied MUC1 DNA vaccine, by preparing the Man-PLL-DNA complex (Tang et al., 2008). The resulting ox-Man-PLL-MUC1 DNA generated immune responses in C57BL/6 mice and protected mice in tumor challenge with a low immunization dose. In addition, the vaccines generated strong immune responses in MUC1 transgenic mice, which are tolerant toward human MUC1 as in humans. Similar to previous reports, the ox-Man-PLL-MUC1 DNA mainly generated a Th1 response while red-Man-PLL-MUC1 DNA generated a Th2 dominant response. A more detailed study showed the differences between DNA alone and Man-PLL-DNA complex upon immunization (Tang et al., 2009). Man-PLL protected cargo DNA against the DNase digestion. Ox-Man and red-Man induced different cytokine secreting profiles. Compared to DNA alone, ox-Man induced higher levels of IL-2, IL-12, IFN-γ, and TNF-α while red-Man induced only IL-2. The Man-PLL-DNA complex was able to stimulate DC maturation through a TLR2 but not a TLR4 dependent pathway.

### Mannan as the Carrier for Allergy Vaccine

Allergen-specific immunotherapy has attracted researchers' attention as it may provide a long-lasting relief from allergy for the patients. Mannan-allergen conjugates have been studied as potential anti-allergy vaccines (Benito-Villalvilla et al., 2018).

The Weiss lab studied the conjugation between oxidized mannan and model allergens, OVA protein and papain, for vaccination targeting dendritic cells (Weinberger et al., 2013). The mannan backbone here served as not only a targeting molecule toward the C-type lectin receptor (a receptor expressing on DCs), but also a platform to induce cross-linking for multimerization of allergen proteins for immunogenicity enhancement (Chackerian et al., 2002). Sodium periodate was used for generating aldehyde groups on mannan backbone for allergen conjugation by oxidative cleavage between C2 and C3. The conjugation efficiency depended on antigen properties as well as the degree of oxidation. The C-lectin binding property of mannan was not disturbed after conjugation with antigen proteins when the oxidation degree was careful controlled. The mannan-antigen conjugate significantly increased the number of antigen-presenting DCs in lymph nodes *in vivo*. Immunization successfully reduced the enzymatic activity or IgE binding capacity of antigen proteins in vaccinated mice. Antibody class-switching from allergy-promoting IgE subtype to non-allergic IgG1 subtype was noticed indicating an anti-allergy therapeutic effect.

Palomares et al. used another strategy to conjugate allergen proteins to non-oxidized mannan by a simple treatment of glutaraldehyde (Scheme 3C) (Manzano et al., 2016). The conjugate took advantage of the trace amount of mannan protein on mannan backbone. Allergens were polymerized and linked to mannan protein through glutaryl diimine linker and the resulting conjugate significantly reduced IgE binding activity against the allergens. Later Palomares et al. applied this conjugation method for preparing P pratense pollen-non-oxidized mannan conjugate (PM) (Sirvent et al., 2016). The PM was hypoallergenic with low IgE binding *in vitro* and induced fewer mast cells under the skin in an *in vivo* skin-prick test. Immunization of rabbit with PM induced blocking antibodies against IgE binding. Compared to the free allergen or the polymerized allergen, the PM can be captured more effectively by human DCs. More anti-inflammatory cytokines IL-6 and IL-10 secretion in human DCs were induced by PM, and Foxp3+ T_reg_ generation through PD-L1 in human subjects was also promoted, which indicated a down-regulation of immune responses toward the allergen.

A drawback in using oxidized mannan is that the mannose ring in the mannan backbone is partially opened, which may impair the capture of PM by DCs in mice and human subjects (Sirvent et al., 2016). This can be overcome with non-oxidized mannan.

Another important consideration in mannan based vaccine is the combination of external adjuvant. In a recent study, the Palomares lab reported the PM induced anti-allergy Foxp3+ T_reg_ generation can be inhibited when co-administrated with Alum (Benito-Villalvilla et al., 2019). This was because Alum suppressed the increasing production of lactate and consumption of glucose induced by PM in human DCs by altering the glucose metabolic fate in mitochondria and inhibiting mammalian target of rapamycin (mTOR).

## α-Galactosylceramide (α-GaLCer)

**Scheme 4:**
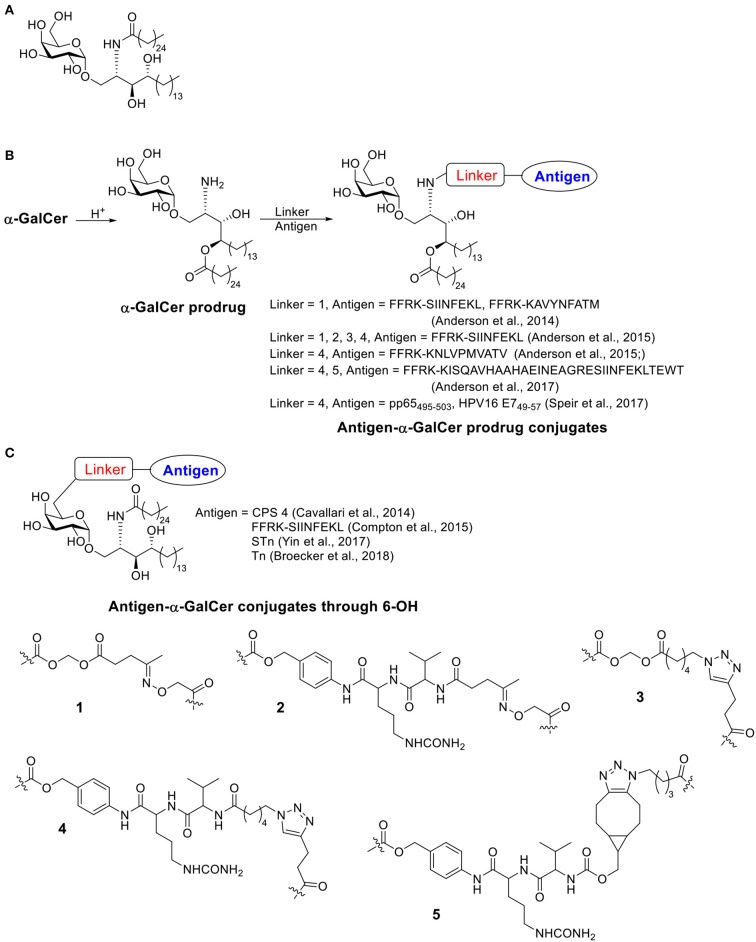
**(A)** Structure of α-GalCer. **(B)** Examples of antigen-α-GalCer prodrug conjugates (conjugate through α-GalCer lipid chain). **(C)** Examples of antigen-α-GalCer conjugate through 6-OH.

The presentation of antigen fragments on antigen presenting cell (APC) surface is an important step for activating the adaptive immune system. Besides the commonly known MHC I and MHC II, CD1 family is a third subset of antigen presenting molecules (Zajonc, 2016). There are 4 types of CD1 (CD1a-CD1d) capable of binding and presenting glycolipids to CD1-restricted T cells. A subtype of T cells, invariant natural killer T (iNKT) cells, is defined as a T cell lineage expressing NK cell receptors and an additional invariant CD1d restricted αβ-T cell receptor (TCR) (Bendelac et al., 2007). After activation through its TCR binding with glycolipid presenting CD1d on APCs, iNKT cells can secret various cytokines, which build a bridge between the innate and the adaptive immune system. iNKT cells can initiate “T dependent (TD) type II response,” which needs no participation of CD4^+^ T cells. It has been reported that iNKT cells play a role in protection against pathogens as well as cancer (Kawano et al., 1997; Metelitsa et al., 2001; Merle et al., 2015). The first iNKT activator, α-GalCer (KRN7000, Scheme 4A) was a synthetic compound discovered from a class of glycolipids originally isolated from marine sponges (Natori et al., 1993; Morita et al., 1995; Shimosaka, 2002). Since then, hundreds of analogs were synthesized by varying the amide side chain length and functional groups, substitutions at galactose-6 position and galactose-ceramide linker etc. α-GalCer is by far the most explored structure and the C-glycoside analog 7DW8-5 with an aryl side chain were also attractive structures for immune studies. Many excellent reviews about α-GalCer and its analogs have been published (Carreño et al., 2014; Marzabadi and Franck, 2017; Waldowska et al., 2017; Zhang et al., 2019).

α-GalCer has been applied as an adjuvant in many studies (Mattarollo and Smyth, 2013; Faveeuw and Trottein, 2014; Artiaga et al., 2016; Liu and Guo, 2017; Fujii et al., 2018; Sainz et al., 2018; Yamashita et al., 2018; Zhang et al., 2019), including vaccines against cancer, influenza, and malaria. To improve the delivery efficiency of α-GalCer and therefore enhancing the activation of iNKT cells, various delivery systems have been designed, such as liposomes, poly(lactic-co-glycolic acid) (PLGA) particles and bacteriophage particles (Macho-Fernandez et al., 2014; Dölen et al., 2016; Ghinnagow et al., 2017a,b; Sartorius et al., 2018). By delivering covalently conjugated antigen and α-GalCer, the immune response could be stronger due to the simultaneous delivery of the antigen and the adjuvant to the same immune cell, and we focus on examples of covalent conjugate vaccines of α-GalCer.

The first examples of covalent conjugation of the antigen and α-GalCer were reported in 2014 (Anderson et al., 2014; Cavallari et al., 2014). The Painter and Herman's lab developed self-adjuvanting vaccines that suppress allergy by conjugating the antigen peptide to α-GalCer through a cleavable linker (Scheme 4B) (Anderson et al., 2014). Starting from α-GalCer, an N to O acyl migration occurred under acidic conditions, which produced an α-GalCer prodrug with a free amino group for further functionalization. The amino group was then capped with an esterase-labile acyloxymethyl carbamate group. The resulting ketone group could be functionalized with an aminooxy peptide containing the protease cleavable FFRK sequence following the desired antigen peptide. Under the physiological condition, the FFRK linker would be cleaved to release the desired antigen while the acyloxymethyl carbamate group would be degraded by an esterase to release the α-GalCer prodrug. After a reversed O to N acyl migration, the active adjuvant α-GalCer would be formed *in situ*. In this study, two model antigen peptides, SIINFEKL and KAVYNFATM, were selected. Both peptide-GalCer conjugates stimulated greater CD8^+^ T cell proliferation compared to non-conjugated mixtures containing the same amount of peptide and α-GalCer. By intracellular staining, large amounts of IFN-γ and TNF-α were detected, while allergy related IL-4 cytokine was not detectable. The conjugates induced antigen-specific cytotoxic responses in immunized animals, while the admixture of peptide and α-GalCer failed to do so. This strong activity was CD4^+^ T cell independent and the covalent conjugation was shown to be critical. The SIINFEKL-α-GalCer conjugate strongly reduced inflammatory responses in an allergy animal model, sensitized by the OVA protein. In contrast, the mixture of peptide and α-GalCer did not reduce the allergic response.

About the same time, the De Libero' lab developed a semisynthetic vaccine against *S. pneumoniae* by conjugating *S. pneumoniae* serotype 4 capsular polysaccharides (CPS 4) to 6 position of α-GalCer through a cleavable linker (Scheme 4C) (Cavallari et al., 2014). Different from Painter and Herman's strategy, the immunogenic lipid tail was kept intact. Instead, an amino moiety was connected to 6-OH of α-GalCer then conjugated with CPS 4 via cyanogen bromide chemistry. The conjugates were usually a mixture of isoureas, *N*-substituted imidocarbonates and *N*-substituted carbamates, which could release the original CPS 4 under acidic condition when taken up by APCs. The CPS 4-GalCer conjugation generated polysaccharide-specific IgM, IgG1, IgG2a, IgG2b, and IgG3 antibody responses in mice, while the mixture of CPS 4 and α-GalCer and CPS 4 only generated weak IgM responses with no IgGs. The conjugation induced germinal centers and the resulting antibody induced *S. pneumoniae* opsonization. Animals vaccinated with the CPS 4-GalCer conjugate exhibited a significant survival advantage (89%) in bacterial challenge model compared to animals receiving CPS alone (25%). By FACS analysis of the splenocytes, CPS 4-GalCer, but not mixture of CPS 4 and α-GalCer or CPS4 alone, induced antibody isotype switching to IgG, generation of memory B cells and antigen secreting plasma cells. Experiments on CD1d^−/−^ mice indicated that iNKT cells were required to establish effective protections against *S. pneumoniae*.

Both conjugation methods, i.e., conjugating antigen to lipid tail or to 6-OH on galactose through cleavable linker, were proven to be successful. The conjugated vaccines have been demonstrated to provide stronger immune stimulation compared to a simple mixture of antigen and adjuvant. Several more examples using either conjugation method have been published since then (Schemes 4B,C).

Painter and Herman continued the study on conjugation linkers and designed several possible linkage methods to covalently conjugate the antigen with α-GalCer (Scheme 4C) (Anderson et al., 2015; Compton et al., 2015). They first investigated four different linkers to link short peptide antigens on GalCer lipid tail (Anderson et al., 2015). Similar to their previous work (Anderson et al., 2014), an *N* to *O* migration of the acyl group on α-GalCer was designed, resulting in an α-GalCer prodrug with a free amino group. The amino group was further capped with an esterase sensitive acyloxymethyl carbamate linker containing ketone (linker **1**) or azido group (linker **3**), or with protease sensitive valine-citrulline-p-amino-benzyl (VC-PAB) carbamate linkers containing ketone (linker **2**) or azido group (linker **4**). Short peptide antigens with a protease cleavable FFRK sequence were conjugated to the 4 different linkers through oxime formation (for linkers **1** and **2**) or copper catalyzed azido-alkyne coupling (CuAAC) (for linkers **3** and **4**). All four conjugates showed similar levels of NKT cell activation in a melanoma challenge model. These conjugates showed improved protection compared to unconjugated mixtures. Among the four choices, linker **4** provided a better stability under physiological pH and eased the synthesis of peptide payload, and therefore was considered as a lead compound for further development.

Painter and Turner applied the aforementioned conjugation strategy for the development of an influenza vaccine. They linked a synthetic long peptide (SLP) containing an immunogenic sequence OVA_257_ (amino sequence: SIINFEKL), a known CD4^+^ T cell epitope OVA_323_ (amino acid sequence: ISQAVHAAHAEINEAGR) and a protease cleavage sequence FFRK, with the α-GalCer prodrug with VC-PAB linker through CuAAC (linker **4**) or strain-promoted alkyne-azide cycloaddition (SPAAC) (linker **5**) (Anderson et al., 2017). Though the two conjugation methods introduced slightly different linker structures in the final α-GalCer prodrug-SLP conjugates, the two vaccines primed NKT cells similarly *in vivo*. As the SPAAC strategy provided a higher yield with fewer side-products, this form of vaccine was subjected to further studies. It has been noted that the α-GalCer prodrug-SLP conjugate vaccine induced CD8+ T memory cell at a similar level as A/PR8-OVA challenged group, which was known to induce OVA specific memory response. The memory T cell response lasted for at least 60 days after immunization. The α-GalCer alone, SLP alone or α-GalCer + SLP mixture failed to induce such memory T cell response. *In vivo* challenge study using OVA modified influenza virus showed that mice vaccinated with the α-GalCer prodrug-SLP conjugates showed a faster viral clearance and body weight recovery compared to α-GalCer alone or α-GalCer + SLP mixture, suggesting the generation of protective immunity by vaccination.

Weinkove and Painter reported an α-GalCer prodrug conjugated with pp65_495−503_, an HLA-A^*^02-restricted peptide from cytomegalovirus (CMV) pp65 protein, through the VC-PAB linker using CuAAC chemistry (linker **4**) (Speir et al., 2017). The resulting conjugate activated human DCs and CD8^+^ T cells besides NKT cells *in vitro*. After incubating human peripheral blood mononuclear cells (PBMCs) with α-GalCer or α-GalCer-pp65_495−503_ conjugate, increased NKT proliferation and IFN-γ secretion were observed. Human DCs can be activated by α-GalCer or α-GalCer-pp65_495−503_ conjugate only when co-cultured with NKT cells. The activation of NKT cells and DCs can be blocked by anti-CD1d antibodies, which suggested α-GalCer-pp65_495−503_ activate human immune cells through the CD1d dependent pathway. The activation of human CD8^+^ T cells also required NKT cells. The conjugation between antigen peptide and α-GalCer is crucial for CD8^+^ T cell activation, as the admixed components failed to induce the expression of T cell activation marker CD137. An oncogenic viral antigen HPV16 E7_49−57_ was conjugated to α-GalCer prodrug through the same strategy and the resulting conjugate vaccine showed significant antitumor response against HPV16 E7 expressing tumor in mice model, which further suggested the effectiveness of α-GalCer prodrug-peptide antigen conjugate strategy.

Painter and Herman's labs also investigated the conjugation of antigen to 6-OH position of α-GalCer through a disulfide bond or a maleimido-linker (Compton et al., 2015). 6″-Deoxy-6″-thiol-α-GalCer was first synthesized and was proven to have similar bioactivities as α-GalCer. The thiol group may be trapped with 2,2′-dithiodipyridine followed by reacting with Cys-peptide to form disulfide bond, or reacting with *N*-propargyl bromomaleimide followed by CuAAC for conjugation with the peptide. Both conjugates induced a stronger peptide-specific cytotoxic response *in vivo* relative to a mixture of α-GalCer and the peptide.

Liu and Guo designed a fully synthetic cancer vaccine candidate by linking tumor associated STn antigen to α-GalCer through a covalent linker at the 6-OH position (Yin et al., 2017). Previous study showed that PEGylation on 6-OH position of α-GalCer through the amide linker retained the specificity of CD1d receptor and the ability to activate iNKT cells (Ebensen et al., 2007). Therefore, the 6 position of α-GalCer was selected as the site of conjugation *via* an amide bond to a non-cleavable linker consisted of a non-branched aliphatic chain to link with the STn antigen. STn-β-GalCer was also synthesized as a weak iNKT activator. The synthetic STn-α-GalCer and STn-β-GalCer were mixed with other lipids to form liposomal vaccines, respectively. Based on ELISA results, though the two vaccines generated similar sera IgM titers against STn on BALb/c mice, STn-α-GalCer induced 23-fold higher IgG titers compared to STn-β-GalCer. Subtype analysis indicated the IgG antibodies were primarily IgG1 and IgG3, which were strong inducers of complement-dependent cytotoxicity (CDC) and antibody dependent cell-mediated cytotoxicity (ADCC). In this case, α-GalCer served as a liposomal carrier as well as an adjuvant for iNKT cell activation. In a later study from the Seeberger lab, the liposomal form of Tn-α-GalCer conjugates showed effective activation of anti-Tn immunity *in vivo* (Broecker et al., 2018). Compared to Tn-CRM_197_, a protein carrier-based vaccine, the anti-Tn IgG response generated by the liposomal form of Tn-α-GalCer conjugate was more consistent and more specific. Furthermore, the liposomal form of Tn-α-GalCer conjugates also generated long-lasting memory response against Tn, while the Tn-CRM_197_ only induced memory response to the carrier protein in some of the mice but not to the glycan antigen. Liposomes formed by Tn-lipid conjugate without the α-Gal structure could also generate anti-Tn IgG, but with a lower magnitude of response compared to Tn-α-GalCer liposomes. The size of the liposomes was shown to be crucial in this case. While the ~400 nm sized liposomes promoted Th1-type IgG2a antibodies, the smaller particles (~120 nm) mainly induced the production of Th2-type IgG1 antibodies. This report indicated the multivalent display of antigens by the antigen-α-GalCer conjugated liposome can be beneficial.

The aforementioned examples have shown the promises of antigen-α-GalCer conjugates as vaccines. The conjugates have been reported to have a stronger protective effect compared to the antigen and α-GalCer mixture. Short peptides and carbohydrates antigens can be used and multiple methods for conjugation were developed, which provided flexible ways for vaccine design. The liposomal form of antigen-α-GalCer covalent conjugates can further help inducing strong and tunable immune responses.

## Modified Dextran

Dextran is a branched natural polysaccharide containing α-1,6-linkage between glucoses as the backbone with α-1,3 linked branches. It is a biocompatible, biodegradable and FDA proved material. Dextran is water soluble and is easy to modify with other functional groups to achieve environment responsive properties. Though crystalized dextran particles can serve as vaccine delivery vehicle as reported (Schröder and Ståhl, 1984; Shen et al., 2013), most studies have focused on modified dextran as a candidate for vaccine design. In this section, we discuss only modified dextran.

### Acetalated Dextran

Acetalated dextran (Ac-Dex) is a pH responsive material first reported in 2008 by the Fréchet's group (Bachelder et al., 2008). It can be synthesized easily from dextran through a single step acetal formation with 2-methoxypropene. In contrast of dextran, Ac-Dex is not soluble in water and can form microparticles using an emulsion procedure. Under acidic conditions, the acetals get hydrolyzed to unmask the parent water soluble dextran structure and therefore breaking up the hydrophobic microparticles. In their study, a model hydrophobic payload, OVA, was encapsulated inside Ac-Dex particles via double emulsion with a loading rate of 3.6 wt%. At pH = 7.4, the particles were stable, while in pH = 5.5 buffers, the particles degraded within 24 h. T cell activation assay showed that OVA loaded Ac-Dex particles significantly increased MHC I presentation of SIINFEKL on RAW macrophages compared to free OVA group. The Huang group applied Ac-Dex to deliver foreign antigens for anti-tumor therapy (Kavunja et al., 2017). The SIINFEKL loaded Ac-Dex particles significantly enhanced the efficiency of SIINFEKL reaching tumor tissue and successfully slowed down tumor growth. *In vivo* CTL assay indicated the OVA loaded Ac-Dex induced CTL responses without additional adjuvants (Kavunja et al., 2017). With the ability of enhancing CTL activation, these Ac-Dex particles were highly efficacious in protecting mice from tumor induced death.

**Scheme 5:**
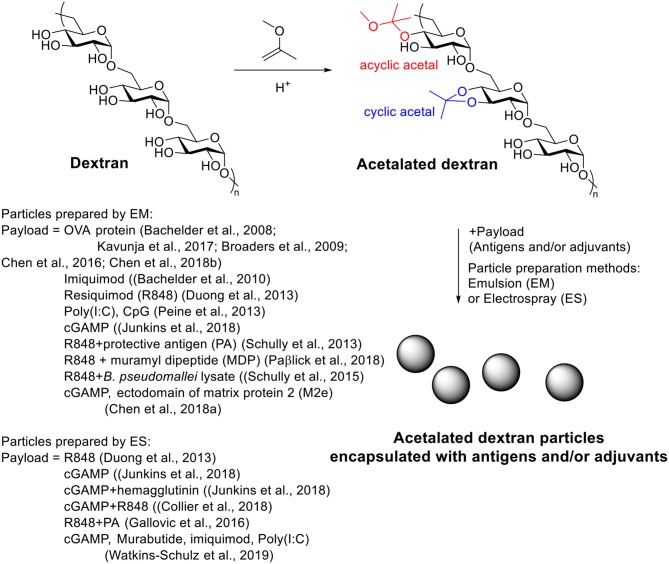
Synthesis of antigen and/or adjuvant loaded acetalated dextran particles.

A great advantage of Ac-Dex over traditional PLGA is the ease in tuning rate of degradation, providing the possibility to optimize the payload releasing rate for a specific application (Broaders et al., 2009; Chen et al., 2016). During the acetal modification, two types of acetal, cyclic acetal which hydrolyzes more slowly and acyclic acetal with faster degradation rates, would be formed on dextran (Scheme 5). As the kinetic product acyclic acetal forms first before the more stable cyclic acetals, the ratio of cyclic/acyclic acetal on the dextran backbone can be tuned by reaction time. The ratio of cyclic and acyclic acetal in the final product dictates the degradation behavior of the Ac-Dex particles. By controlling the reaction time from 2 to 1,500 min, a set of Ac-Dex with different ratios of cyclic/acyclic acetal was prepared (Broaders et al., 2009). The degradation half-life at pH = 5.5 was tuned from minutes to days. The degradation rates at pH = 7.4 were usually 230–280 times slower than those at pH = 5.5, which was stable enough for delivery applications. Half-life of degradation correlated well with cyclic acetal content, which indicated the hydrolysis of cyclic acetal may be the rate-limiting step in particle degradation. The molecular weight of dextran also influenced the degradation of particles (Chen et al., 2016). With similar cyclic acetal coverage, the Ac-Dex with higher molecular weight degraded faster.

The degradation rate can be important for both MHC I and MHC II antigen presentation (Broaders et al., 2009). OVA loaded Ac-Dex particles with degradation half-lives from 0.27 to 16 h were prepared and incubated with bone marrow dendritic cells (BMDCs) followed by T cell activation assays to determine MHC I and MHC II presentation of OVA derived epitopes. The particles with 1.7 h degradation half-life led to an optimal MHC I or MHC II presentation of OVA derived epitopes compared to particles with either longer or shorter degradation half-life. These optimal particles performed an order of magnitude better than traditional PLGA or iron oxide particles. Interestingly, the Ac-Dex particles with 1.7 h degradation half-life did not require the transporter to be associated with antigen processing (TAP), a protein involved in the most common MHC I antigen loading mechanism, for antigen presentation, while the particles with 16 h degradation half-life required TAP for antigen loading (Broaders et al., 2009). The difference might be attributed to the surface chemistry difference of the two materials due to the different degradation rate. A recent *in vivo* study (Chen et al., 2018b) showed that OVA loaded Ac-Dex particles with 20% cyclic acetal coverage (CAC) generated stronger antibody response during the entire experiment period compared to particles with 40 and 60% CAC. Notably, when the particles were used for adjuvant delivery, the immune activating behavior was different. The adjuvant loaded Ac-Dex particles with 20% CAC induced stronger antibody and cytokine response at early time points (day 14), while the 40 and 60% CAC induced greater antibody titers at later time points (days 28 and 42). This study suggested the importance of delivery of antigen and adjuvant separately in individually optimized Ac-Dex particles.

One possible limitation for Ac-Dex is that, one of the products released from degradation is methanol, which is known to be highly toxic. Therefore, 2-ethoxypropene was explored as an alternative to functionalize dextran instead of 2-methoxypropene (Kauffman et al., 2012). No significant differences were observed in cell viability when cells were incubated with the acetalated dextran formed with 2-ethoxypropene or Ac-Dex at concentrations below 1 mg/ml. Further toxicity study is needed to determine if the new acetalated dextran improved the biocompatibility at higher concentrations. To date, most studies have been using Ac-Dex as the carrier material.

Ac-Dex has been introduced for vaccine adjuvant delivery since 2010 (Bachelder et al., 2010) for several types of TLR agonists. Keane-Myers and co-workers first studied Ac-Dex microparticles as the delivery platform for imiquimod, a hydrophobic TLR7/8 agonist, as an adjuvant *in vitro*. Imiquimod loaded Ac-Dex microparticles were prepared with 4 wt% loading rate and 100% loading efficiency. After incubation with imiquimod loaded particles, the gene expression level, cytokine secretion level of inflammatory cytokines IL-1β, IL-6, and TNF-α, and the expression of two activation markers PD1-L1 and iNOS as well as the production of downstream product NO, were significantly increased in two macrophage cell lines, MH-S and RAW 264.7. The particles also significantly increased the production of IL-1β, IL-6, IL-12p70, and MIP-1α in BMDCs. Compared to free imiquimod, the encapsulated imiquimod induced higher amounts of cytokine at lower concentrations of the particles. Empty Ac-Dex did not induce detectable inflammatory cytokine or activation marker increases. This *in vitro* study showed the promise of Ac-Dex as a vaccine adjuvant carrier to achieve a good immune stimulation effect.

Another method for Ac-Dex particle preparation, electrospray (ES), provided a better encapsulation efficiency (83%) toward a less hydrophobic TLR 7/8 agonist resiquimod compared to the standard emulsion encapsulation method (6%) (Duong et al., 2013). Particles made by electrospray were larger (1–5 μm) than those from the emulsion method (~300 nm) and had a collapsed morphology. More spherical particles could be obtained when blending with Tween 80 during electrospray process. The Tween 80-blended Ac-Dex particles stimulated macrophages *in vitro* to increase NO release and inflammatory cytokine secretion. The *in vivo* study showed that these particles reduced *L. donovani* amastigotes in heart and liver of mice relative to mice receiving empty nanoparticles or PBS.

The Ainslie's group applied Ac-Dex to deliver another two TLR agonists, i.e., poly I:C and CpG as vaccine adjuvants (Peine et al., 2013). 71 kDa Ac-Dex with 5 min acetalation reaction time [Ac-Dex (5min)] was found to be the best material for the delivery of both agonists. The encapsulation efficiencies of poly I:C and CpG in Ac-Dex (~55 and ~36%, respectively) were significantly higher compared to traditional PLGA particles (~33 and ~3%, respectively). A significantly higher level of NO release and cytokine secretion including IL-6, IL-12p70, IL-1β, IL-2, TNF-α, and IFN-γ was observed in RAW 264.7 macrophages with poly I:C encapsulated Ac-Dex (5 min) particles compared to poly I:C encapsulated PLGA particles and another Ac-Dex, Ac-Dex (4 h), which degraded slower. Due to the poor encapsulation of CpG in PLGA (~3%), only Ac-Dex (5 min) was tested for delivering CpG to RAW 264.7. For both NO release and cytokine profile, CpG encapsulated in Ac-Dex was superior to free CpG.

Ting's lab applied Ac-Dex particles for the delivery of cyclic dinucleotide (CDN) 3′3′-cGAMP, a ligand of stimulator of interferon genes (STING), for immune cell activation (Junkins et al., 2018). The cGAMP is a water-soluble adjuvant, which has poor cell penetration abilities. Liposomes and hydrogel delivery carrier of cGAMP were associated with low encapsulation efficiency and poor long-term stability (Hanson et al., 2015; Irvine et al., 2015; Lee et al., 2016; Koshy et al., 2017). With the electrospray method, the Ac-Dex particles (ES Ac-Dex) loaded up to 0.52%wt of cGAMP with 89.7% encapsulation efficiency, which is significantly higher compared to Ac-Dex particles prepared through the emulsion method (EM Ac-Dex), PLGA particles or liposomes. The cGAMP loaded ES Ac-Dex remained intact in pH neutral media at 37°C for at least 28 days without losing the bioactivity of cGAMP. Strong immune activation was observed both *in vitro* and *in vivo* without significant toxicities. When ES Ac-Dex was co-administrated with a model antigen OVA, the level of antibody against OVA generated *in vivo* was enhanced by 10^4^ to 10^6^ folds compared to OVA alone. Analysis of antibody subtype indicated the cGAMP encapsulated ES Ac-Dex particles induced balanced Th1 and Th2 associated immune responses, while the Alum adjuvant produced mainly Th2 polarized responses. Besides humoral responses, the cGAMP encapsulated ES Ac-Dex also induced cellular responses against the model antigen OVA. On a B16F10 melanoma model, the cGAMP Ac-Dex showed a better anti-tumor effect compared to three other Ac-Dex particles encapsulating different adjuvants, Murabutide, imiquimod, and Poly I:C (Watkins-Schulz et al., 2019). The successful anti-cancer immunotherapy by cGAMP Ac-Dex particles was also observed on a triple negative breast cancer cell line E0771. Systematic administration of cGAMP Ac-Dex through intravenously injection slowed down tumor growth as efficient as local administration through intratumoral injection. Interestingly, in the B16F10 model, the NK cells, instead of T cells, were the major type of cells for tumor lysis. However, for E0771 tumor, both NK and T cells were important for the anti-tumor responses. These results indicated the importance of activating both the innate immune cells (NKs) and adaptive immune cells (T cells) for tumor immunotherapy, as the T cells may not always be the major anti-tumor responders.

Co-delivering more than one adjuvant within one Ac-Dex particle can improve the immune activation compared to single adjuvant loaded Ac-Dex particles. For example, cGAMP ES Ac-Dex successfully induced high levels of IFN-β, IL-6, and TNF. With the co-encapsulation of resiquimod (R848) in the same particle, the cGAMP/R848 ES Ac-Dex elicited two more important cytokines for adaptive immune activation, IL-1β, and IL-12p70 (Collier et al., 2018). Co-administration of separate cGAMP ES Ac-Dex and R848 ES Ac-Dex particles was not as efficient as co-encapsulation of the two adjuvants within the same particle based on *in vitro* cytokine release study. The combination of muramyl dipeptide (MDP), a NOD2 ligand, with R848, also showed superior additive effects (Paßlick et al., 2018).

Besides serving as an adjuvant carrier, Ac-Dex particles can deliver both the antigen and the adjuvant as a full vaccine against various targets, such as anthrax, bacterial infection and influenza.

Anthrax caused by the infection of (*B. Anthracis)* can lead to death within 1 week, with the current vaccine Anthrax Vaccine Adsorbed requiring up to 6 doses and 18 months to achieve protection (Schully et al., 2013). A vaccine that can generate fast immune protection against anthrax is urgently needed. The Ainslie' group designed a Ac-Dex based vaccine to generate a rapid immune response against anthrax, where Ac-Dex was used to encapsulate R848, and Protective Antigen (PA), the most important toxic component of anthrax antigen, in separate particles by emulsion (Schully et al., 2013). Mice received both R848 Ac-Dex and PA Ac-Dex showed much stronger IgG responses on days 14, 28, and 42 after immunization compared to PA+Alum or free PA + R848 Ac-Dex particles. All mice immunized with PA Ac-Dex +R848 Ac-Dex vaccine survived 3 challenges on days 14, 28, and 42 with both low and high doses of *B. Anthracis*. This Ac-Dex based vaccine only required two injections at days 0 and 7, and effective protection against anthrax was observed as early as 14 days. The fast generation of protective immune response by Ac-Dex based vaccine provided a promising way fighting against fast progressing diseases. In a later study, electrospray method was used instead of emulsion to fabricate Ac-Dex particles with PA only or with both PA and R848 (Gallovic et al., 2016). Three vaccine formulations were used to immunize the mice: (i) PA absorbed to resiquimod microparticles; (ii) PA and resiquimod encapsulated in separate particles; and (iii) PA and resiquimod encapsulated in same particle. Both (ii) and (iii) induced high IgG1 and IgG2a titers on day 42 after immunization similar to or higher than Anthrax Vaccine Adsorbed, the current anthrax vaccine. The *in vivo* study showed that (ii) was the best vaccine, which protected 50% mice from death during 28-days observation, while mice immunized with (iii) only had 10% survival. BioThrax group did not survive beyond 13 days. The *in vivo* study indicated that delivering PA and adjuvant in separate particles may provide a faster and stronger immune response toward anthrax. This finding supported the idea that adjuvant and antigen should be encapsulated in separate Ac-Dex particles optimized for each component with different CAC percentages (Chen et al., 2018b).

Ac-Dex was used as carrier for a *Burkholderia pseudomallei* subunit vaccine and showed the ability to generate immune responses within a short time period (Schully et al., 2015). The antigen *B. pseudomallei* lysate and an adjuvant R848 were encapsulated in separate Ac-Dex particles. The rapid immunization schedule (two injections on day 0 and 7) slowed down the death progress during 26-days observation when mice were challenged on day 14 with a lethal dose *B. pseudomallei*. 12% of the immunized mice survived the challenge on day 26 while most mice in control groups died within 2 days of challenge and none survived beyond 20 days. The vaccinated group had higher antibody titers, stronger cytokine secretion (IL-4, IL-5, IL-17A, IL-12, IFN-γ, GM-CSF, and TNF-α) and more cytotoxic T cells compared to the control group receiving PBS only.

The Ting lab applied the cGAMP encapsulated Ac-Dex with soluble hemagglutinin (HA) protein from H1N1 influenza virus for anti-influenza vaccination (Junkins et al., 2018). A strong Th1-biased antibody response was observed in cGAMP Ac-Dex + HA group, while Alum + HA only induced weak Th2-biased antibody response. The cGAMP Ac-Dex + HA protected 12 out of 13 mice from H1N1 influenza challenge, while >90% of untreated mice and >75% of mice immunized with free HA only were killed during the challenge. The neutralizing antibodies generated by cGAMP Ac-Dex + HA remained detectable in mouse sera for more than 4 months after immunization and protected the mice from a lethal dose of H1N1 influenza virus challenge 7 months after immunization. The Bachelder lab investigated the co-administration of cGAMP Ac-Dex and the ectodomain of matrix protein 2 (M2e) encapsulated Ac-Dex particles as an anti-influenza vaccine (Chen et al., 2018a). The M2e and cGAMP were encapsulated in separate Ac-Dex particles with different percentage of CAC. In contrast to the delivery of OVA antigen where a high antibody titer was observed in Ac-Dex particles with low CAC (20%) (Chen et al., 2018b), it was observed that the M2e Ac-Dex with high CAC (60%) induced higher antibody titers compared to M2e Ac-Dex with lower CAC (40 and 20%). The cGAMP encapsulated Ac-Dex particles with different CAC (20, 40, or 60%) did not significantly change the antibody titers. The M2e and cGAMP encapsulated in separate Ac-Dex particles (60% CAC) induced significantly higher antibody titers compared to the co-encapsulation of M2e and cGAMP in same Ac-Dex (60% CAC). Besides the antibody titer, significantly higher levels of IFN-γ, IL-2, and IL-6 secretion were detected in mice immunized with M2e Ac-Dex (40% or 60% CAC) + cGAMP Ac-Dex (60% CAC), which suggested a successful generation of cellular immunity. Both vaccines, M2e Ac-Dex (40%) + cGAMP Ac-Dex (60%) and M2e Ac-Dex (60%) + cGAMP Ac-Dex (60%) showed significant improvement of survival during a lethal dose influenza challenge in mice.

The studies discussed so far relied on passive uptake of the Ac-Dex particles by immune cells. The Fréchet group studied mannosylated Ac-Dex particles for immunomodulation through mannose targeting (Cui et al., 2011). “Click-able” Ac-Dex was obtained by partially modifying the hydroxyl groups on dextran backbone with an azido-triethylene glycol linker followed by acetalation. Microparticles were then prepared through the emulsion method with subsequent surface mannosylation using the CuAAC reaction. These particles (referred to as Man-Ac-Dex) with high density mannose on the surface (up to 10^6^/particle) had high binding avidity to mannose receptors on DC surface. Man-Ac-Dex showed 1.5–2 fold increase of DC uptake and about 5 fold increase of MHC I presentation on DCs compared to Gal-Ac-Dex, azido-Ac-Dex, or Ac-Dex particles, suggesting more potent immune activation. However, no *in vivo* study was performed with particles.

### Reducible Dextran Nanogel

**Scheme 6:**
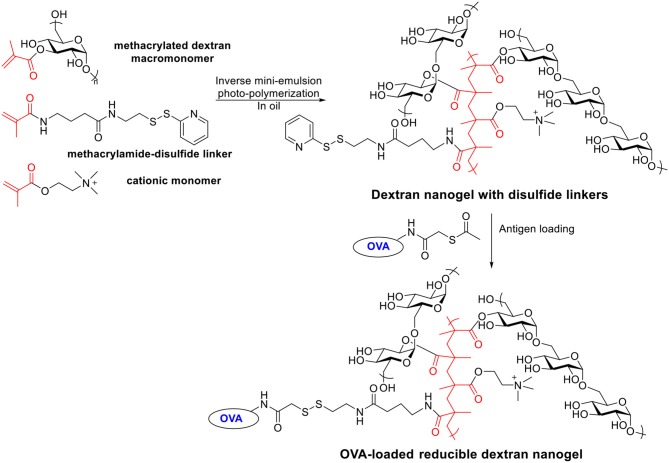
Synthesis of OVA-loaded reducible dextran nanogel.

Besides acetalated dextran, reducible dextran nanogel is another type of modified dextran, which has been developed for antigen delivery to DCs (Li et al., 2015, 2016). A cationic dextran nanogel has been fabricated by inverse mini-emulsion photo-polymerization with methacrylated dextran, a methacrylamide functionalized disulfide linker, and a positively charged methacrylate monomer. The nanogel was then covalently conjugated with a model antigen OVA through a disulfide linker (Scheme 6). The confocal microscopy indicated the OVA conjugated nanogel enhanced the uptake by D1 cells compared to non-covalently loaded OVA-nanogel, free OVA or empty nanogel. The OVA-conjugated nanogel combined with poly I:C significantly slowed down the growth of B16-OVA tumor expressing OVA antigen in a mouse tumor model compared to free OVA, non-covalent OVA-nanogel (Li et al., 2016). A preventive antitumor model was studied by immunizing C57BL/6 mice on days 0 and 14 with different vaccine formula followed by tumor challenge on day 28 with B16-OVA cells. All PBS or empty nanogel treated mice died within 20 days after tumor cell injection. Only 30% of the mice in non-covalent OVA-nanogel group were tumor-free on day 52, while 90% of mice immunized with OVA-conjugated nanogel+poly I:C remained tumor free. OVA-conjugated nanogel+poly I:C induced highest percentage of OVA specific CD8^+^ T cell and OVA specific IgG titers. In addition to the preventive model, the efficacy of the vaccine was investigated in a therapeutic model. Mice were injected with B16-OVA on day 0, which was followed by two immunizations on days 6 and 16. While all other groups developed fast-growing tumor and died within 35 days, the OVA-conjugated nanogel+poly I:C significantly slowed the tumor growth and prolonged the survival.

These two studies showed the reducible nanogel carrier can enhance DC activation *in vitro* and generate significant preventive and curative effects against tumor *in vivo*. It was found that the OVA-loaded nanogel exhibited cytotoxicity at high concentrations, which may require more chemical modifications to improve biocompatibility (Li et al., 2015). For example, the percentage of the cationic monomer may be lowered to reduce the level of positive surface potential to decrease cytotoxicity.

### Oxidation Sensitive Dextran

**Scheme 7:**
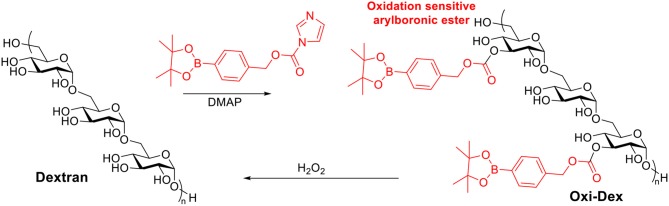
Synthesis of oxidation sensitive dextran.

Reactive oxygen species are heavily produced in the phagosomes of APCs, which are crucial for initiating immune responses (Jones, 2008; Winterbourn, 2008). It has been reported that the most effective APCs, DCs, may have phagosomes with H_2_O_2_ concentration up to 1 mM (Savina et al., 2009). Therefore oxidation sensitive dextran was investigated as a vaccine carrier candidate (Broaders et al., 2011). Free hydroxyl groups on dextran were modified with arylboronic ester resulting in Oxi-Dex (Scheme 7). 100–200 nm sized particles were prepared via the standard emulsion method. The resulting particles were stable in PBS buffer but decomposed in 1 mM H_2_O_2_ with a half-life of 36 min. The OVA encapsulated Oxi-Dex induced a 27-fold increase of OVA presentation in DC 2.4 cells compared to OVA encapsulated PLGA particles, while free OVA did not get presented. However, this Oxi-Dex was not further studied after this report.

### pH Sensitive Amphiphilic Galactosyl-dextran-retinal Conjugates (GDR)

The galactosyl-dextran-retinal (GDR) conjugates is a pH sensitive amphiphilic material reported by the Ma group (Wang et al., 2016). All-*trans* retinal, the precursor of retinoic acid (active metabolite of Vitamin A), was first conjugated to dextran through a pH-responsive hydrazone bond then further modified with ethylenediamine following reaction with NHS activated lactobionic acid to obtain the GDR conjugate. GDR was amphiphilic, which spontaneously self-assembled into nanogel with size around 115 nm and zeta-potential around 27 mV. At pH = 7.4, the GDR was relatively stable with <10% of retinal release within 48 h. However, the hydrazone bond in GDR conjugate could be rapidly cleaved at pH 5.0 resulting in over 50% retinal release within 24 h, which could serve as an adjuvant. GDR nanogel induced BMDC maturation *in vitro* while free retinal failed to do so. OVA-loaded GDR nanogel enhanced both MHC I and MHC II antigen presentation on BMDCs. The release of retinal from GDR nanogel significantly elevated the reactive oxygen species (ROS) generation in BMDCs by 2–3 folds relative to free all-trans retinal within 4 h due to lysosomal disruption, and the resulting ROS significantly enhanced proteasome activity in BMDCs. In a B16-OVA tumor model, the OVA-GDR nanogel vaccine suppressed tumor growth and prolonged mouse survival compared to free OVA, free OVA+retinal and PBS groups. OVA-GDR nanogel induced robust CD 8^+^ T cell proliferation as well as high levels of IFN-γ production and lysis of tumor cells.

## β-Glucans

β-Glucans are β-1,3-linked glucose polymers with β-1,6 branches. β-Glucans can be isolated from fungal cell wall, bacteria, seaweed, cereal, etc. Depending on the source, the polysaccharides may have varied primary, secondary or tertiary structures, or physical properties. Though heterogeneous, these polysaccharides can induce similar immune responses and therefore usually termed as a common name “β-glucans” (Novak and Vetvicka, 2008). The major β-glucan receptors in mammals are dectin-1, and complement receptor 3 (CR3, CD11b/CD18) (Levitz et al., 2015). It has been reported that the stimulation via dectin-1 primes Th1, Th17, and cytotoxic T lymphocyte responses (LeibundGut-Landmann et al., 2008; Geijtenbeek and Gringhuis, 2009; Levitz et al., 2015). With their immune stimulating properties, β-glucans have been studied in vaccine design with an established record of safety in both preclinical and human trials (Williams et al., 1988; Novak and Vetvicka, 2008; Weitberg, 2008). As a major component of fungal cell wall, β-glucans has been widely used as antigens for generating anti-glucan antibodies against fungal infections (Bromuro et al., 2010; Cassone and Casadevall, 2012; Liao et al., 2015). In this section, we focus on examples applying β-glucans as vaccine carriers and built-in adjuvants.

### β-Glucan Particles

β-Glucan particles (GPs) are the most studied vaccine carriers in the β-glucan family. They were developed in 1980s but only widely used as vaccine carriers in recent years (Hunter et al., 2002; Mirza et al., 2017; Abraham et al., 2019). GPs are highly purified, hollow porous cell wall shells with 2–4 μm sizes. GPs can be derived from baker's yeast through a series of hot alkali and organic extractions (Di Luzio et al., 1979; Williams et al., 1989). It contains primarily 1,3-β-glucans along with small amounts of β-1,6-glucans and chitin (Levitz et al., 2015). GPs can be recognized by dectin-1 and upregulate cell surface presentation of MHC molecules and co-stimulation molecules as well as inducing the production of inflammatory cytokines (Hunter and Redelman, 2004; Berner et al., 2005; Huang et al., 2009). The hollow GPs have been studied as carriers for proteins, DNA, siRNA, and other small molecules (Soto and Ostroff, 2008; Aouadi et al., 2009; Huang et al., 2010, 2012; Tesz et al., 2011; Soto et al., 2012).

Antigens can be non-covalently trapped inside GPs with the addition of polymers such as yeast tRNA, alginate-calcium or alginate-calcium-chitosan mixture. The Levitz group used tRNA to trap OVA protein inside GPs (Huang et al., 2010). These GPs were efficiently taken up and proteolyzed by DCs to induce DC maturation. Significant T cell proliferation was observed when incubated with GP-OVA at concentrations starting from 0.03 μg OVA /ml, while the free OVA protein needed 100 times higher concentration to reach similar stimulation levels. The CD4^+^ T cells isolated from GP-OVA immunized mice secreted significantly higher amounts of pro-inflammatory cytokines such as IL-4, IL-17, and IFN-γ compared to Alum/OVA immunized mice. For antibody responses, the GP-OVA vaccine successfully induced Th1 skewing antibody subtype IgG2c, while the Alum/OVA induced only IgG1 responses. The long-term immune responses were monitored 18–20 months after the last immunization (Huang et al., 2013). The CD4^+^ T cells isolated from immunized mice resumed cytokine secretion upon *ex vivo* OVA stimulation, and the serum antibody titer remained detectable. Notably, the encapsulation of OVA in GPs was found important, as the admixture of OVA and GPs was not as effective in inducing CD4^+^ T cell cytokine secretion and antibody responses (Huang et al., 2013). The Levitz group also studied polymers such as alginate-calcium (AC) or alginate-calcium-chitosan (ACC) mixture for trapping antigens in GPs (Huang et al., 2013). The AC and ACC trapped GP-OVA showed comparable capacities to induce antigen-specific T cell responses and antibody responses in mice as the tRNA trapped GP-OVA. Other antigens such as BSA (De Smet et al., 2013), FedF (Baert et al., 2016), could also be trapped inside GPs as vaccine candidates.

Antigens can be loaded into GPs through covalent coupling. The Hunter group covalently conjugated antigen BSA to GPs through amide bonds (Berner et al., 2008). The BSA-GP conjugates were phagocytized by macrophages and both intradermal and oral administration of BSA-GP vaccine induced immune responses against BSA. OVA-GP conjugates were synthesized similarly, which induced strong BMDC, CD4^+^ and CD8^+^ T cells activation *in vitro* (Berner et al., 2015). The Hong group prepared OVA loaded GPs in organic phase, which reduced GP aggregation compared to aqueous phase conjugation, and provided more homogenous OVA-GPs (Yang et al., 2017). With this novel conjugation method, the GPs were first dispersed in cyclohexane/Igepal CO-520 (85:15) solution followed by the addition of aqueous solution containing the OVA antigen and glutaraldehyde cross-linker sequentially. The hydrophilic antigen and cross-linker would be slowly soaked into GP cavity due to the hydrophilic environment of the glucans and the conjugation primarily took place inside the GP cores rather than on the exterior of the GPs, which may cause cross-linking between particles and lead to aggregation. The resulting OVA-GPs successfully induced BMDC maturation and T cell proliferation *in vitro* and stimulated B cell activation and germinal center formation *in vivo*. High anti-OVA IgG2c titers were detected after only one immunization with the OVA-GP vaccine, which indicated a strong Th1 biased immune response. The OVA-GPs successfully induced antigen-specific CD8^+^ T cell response *in vivo* and provided significant protection against tumor development to EG.7-OVA tumor bearing mice.

An interesting property of GPs is that they can be administered orally. GPs can be taken up by human intestinal epithelial cells and induce the secretion of chemokines and the expression of pattern recognition receptors and costimulatory molecules (De Smet et al., 2013). The GP-OVA complex can be delivered by M cells to mucosal lymphoid tissues and induce the proliferation of OVA specific CD4^+^ T cells when given orally to mice. Surface functionalization of an immunoglobulin-binding protein G followed by the anti-aminopeptida N (APN, an intestinal epithelial receptor) antibody on GPs can further enhance the passage of particles through the epithelial barrier (Baert et al., 2015). Compared to isotype antibody conjugated GPs, the anti-APN GPs can be internalized 10 times more by intestinal epithelial cell line IPEC-J2 at a 16-fold lower concentration. *In vivo* study showed that orally administrated anti-APN-coated, FedF-loaded GPs induced significantly higher titers of antibodies compared to non-targeting FedF loaded GPs.

### β-Glucan-antigen Complex

**Scheme 8:**
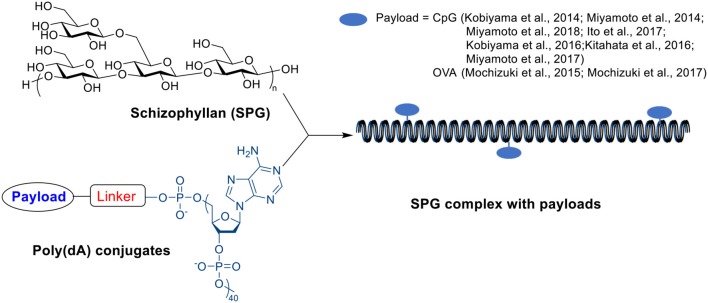
Structure of SPG, Poly(dA) and SPG complex.

A β-glucan member, schizophyllan (SPG), contains a β-1,3-glucan main chain with β-1,6-glycosyl side chain every three glucose residues. It can form stoichiometric complexes with specific homonucleiotides such as poly(C) or poly (dA) via a combination of hydrogen bonding and hydrophobic interactions (Scheme 8) (Sakurai and Shinkai, 2000; Sakurai et al., 2001; Numata et al., 2003). Unlike β-glucan particles, these SPG complexes are nano-rod shaped with diameters around 10–20 nm (Kobiyama et al., 2014). The complex includes two SPG chains and one polynucleiotide chain forming a triple helix through interactions between two main-chain glucoses and one base, and the stability of complex depends on the length of polynucleotide (Sakurai and Shinkai, 2000; Sakurai et al., 2001). The complex can be recognized by dectin-1 receptor inducing immune responses (Minari et al., 2010; Mochizuki and Sakurai, 2011), and therefore have been studied as vaccine adjuvants.

A complex of SPG with CpG-dA_40_, a short single stranded DNA fragment with CpG motif and a 40-mer poly(dA) tail, has shown strong immune activating effects due to the combination delivery of immunocytes targeting SPG and immuno-stimulative CpG (Kobiyama et al., 2014; Miyamoto et al., 2014, 2018). This complex can induce antigen-presenting cell activation as well as Th1 and CD8 T cell responses (Kobiyama et al., 2014, 2016; Ito et al., 2017). Intravenous injection of CpG-SPG complex suppressed tumor growth more efficiently than SPG, CpG or mixture of SPG and CpG on several tumor models (Kitahata et al., 2016). The CpG-SPG complex could be cross-linked to form nanogels with a larger size (~150 nm) by mixing CpG-SPG and its complementary sequence (Kobiyama et al., 2014; Miyamoto et al., 2017), which may further improve the delivery efficiency toward immune cells due to the size effect (Manolova et al., 2008). Compared to CpG-SPG complex, the cross-linked CpG-SPG nanogel induced significantly higher IL-6 secretion in mice splenocytes *in vitro* (Kobiyama et al., 2014). The fluorescence microscopy imaging indicated a 10 times higher uptake of the cross-linked CpG-SPG nanogel than CpG-SPG complex by macrophages (Miyamoto et al., 2017). The CpG-SPG nanogel induced more antigen specific CD8^+^ T cells *in vivo* compared to CpG-SPG complex when co-administrated with OVA antigen. The nanogel immunization significantly slowed down EG7 tumor growth and prolonged survival in mice compared to free CpG or CpG-SPG complex (Miyamoto et al., 2017).

Besides CpG, peptide antigens can be conjugated with poly(dA) for preparing SPG-antigen complexes. The Sakurai group reported an OVA-SPG complex prepared with OVA peptide-poly(dA) conjugate and SPG (Mochizuki et al., 2015, 2017). It was observed that the conjugation strategy could influence the immune cell processing of the OVA-SPA complex (Mochizuki et al., 2017). OVA-poly(dA) conjugated through a glutathione cleavable disulfide linker can induce significantly higher levels of OVA antigen presentation on macrophages compared to the OVA-poly(dA) conjugated through a triazole. The conjugation of poly(dA) at the N terminal of OVA peptide, instead of at the C terminal, showed a higher OVA presentation by macrophages (Mochizuki et al., 2017). The OVA-SPG induced peptide specific CD8^+^ T cell responses both *in vitro* and *in vivo* when co-administrated with CpG-SPG complex. OVA-SPG/CpG-SPG vaccine immunized mice showed significantly more effective *in vivo* lysis of OVA-pulsed target cells compared to free OVA peptide, free OVA + free CpG and free OVA + CpG-SPG group as indicated by *in vivo* CTL assays (Mochizuki et al., 2015). The strong CTL activation was observed with a very low dose of OVA peptide (100 ng/mouse) (Mochizuki et al., 2017). The OVA-SPG/CpG-SPG vaccine also successfully suppressed the growth of EG7 tumor and prolonged survival time in mice (Mochizuki et al., 2015).

### β-Glucan Based Nanoparticles for Vaccine Delivery

Beside the large-sized GPs and rod-shaped SPG complexes, β-glucan nanoparticles were investigated for vaccine delivery. The Dong group developed a synthetic MUC1 vaccine by conjugating MUC1 peptide with a β-glucan chain (Wang et al., 2019). The resulting MUC1-β-glucan material formed homogenous nanoparticles sized 150 nm due to hydrophobic interactions. This MUC1-β-glucan nanoparticle induced significantly higher serum antibody titers and IFN-γ and IL-6 cytokines. The Zhang lab prepared β-glucan nanoparticles based vaccines by mixing positively charged aminated β-glucan with negatively charged CpG adjuvant and OVA protein antigen (Jin et al., 2018). The combination of dectin-1 activating β-glucan and TLR-9 activating CpG in one nanoparticle showed synergistic effects in inducing both strong humoral and cellular immune responses.

The Kono lab reported a set of β-glucan based pH sensitive materials for cytoplasmic delivery of antigen (Yuba et al., 2017). Curdlan, a kind of β-glucan, was modified with methyl glutaric acid (MGlu) to generate a pH responsive polysaccharide MGlu-Curd. Using a similar strategy, pH responsive 3-methyl glutaryl mannan (MGlu-Man), and 3-methyl glutaryl dextran (MGlu-Dex) were prepared. 1-Aminodecane was then conjugated to these polysaccharides to anchor these pH responsive polysaccharide chains onto membranes of OVA-loaded liposomes. All three types of liposomes with different polysaccharides induced the release of cargo from liposome at around pH 5. The polysaccharide backbone played an important role for obtaining liposomes with high affinity to DC cells. Compared to MGlu-Man and MGlu-Dex coated liposomes, the liposome containing MGlu-Curd with 59 MGlu groups per chain (MGlu_59_-Curd), induced the highest DC uptake of the liposomes. The percentage of MGlu modification also influenced the immune activation. In general, curdlan with higher percentage of MGlu content (MGlu_71_-Curd and MGlu_59_-Curd) induced higher pro-inflammatory cytokines such as TNF-α and IL-12 in DC2.4 cells compared to those with lower MGlu content (MGlu_41_-Curd and MGlu_21_-Curd). Compared to MGlu-Man and MGlu-Dex, MGlu_59_-Curd elicited more IFN-γ and higher cell-mediated cytotoxicity in splenocytes isolated from OVA-immunized mice *in vitro*. The tumor challenge study showed that mice immunized with MGlu_59_-Curd had the smallest tumor size and longest survival time highlighting the advantage of the curdlan backbone.

## Conclusions and Future Outlook

In summary, we have reviewed recent advances in vaccine development applying carbohydrates as adjuvants and/or vaccine carriers. With their biocompatibility, ease for modification, and the ability to interact with the immune system through multiple mechanisms, carbohydrates provide a great variety of choices to meet the various needs for vaccine studies.

Carbohydrates can be modified through multiple methods such as amide or ester formation, CuAAC reaction, oxidation of sugar rings followed by imine or oxime formation, which make them flexible for various applications in vaccine designs. For example, the controlled release of the antigen and adjuvant from the vaccine carrier is important for immune activation. A desired carrier should not release their cargos before entering immune tissues, and should not release too slow after encountering immune cells, which may fail to produce enough immune stimulation resulting in tolerance (Lofthouse, 2002; Sivakumar et al., 2011).

The optimal deliveries of antigens and adjuvants can be different, and the carriers may need to be optimized separately (Chen et al., 2018a,b). By controlling the reaction time during the acetalation of dextran, a carbohydrate-based vaccine carrier with fine-tuned releasing profile can be achieved, which can serve as a great platform for vaccine optimization. Antigen-MPLA and antigen-α-GalCer conjugates can be easily combined with other well-studied lipid molecules to form liposomal vaccines. Taking advantage of the well-developed strategies for liposome preparation (Abu Lila and Ishida, 2017; Bulbake et al., 2017), carriers with controlled size and surface charges, another two important factors for immune targeting (Xiang et al., 2006; Bachmann and Jennings, 2010), can be obtained.

Notably, although there are many examples showing that successful carbohydrate conjugate-based vaccines can be achieved through multiple chemistry reactions and linker structures, the small structure alteration of carbohydrate backbones due to the conjugation may significantly influence the final immune outcomes. The carbohydrates often contain more than one position available for chemical modification. When designing carbohydrate vaccines, the conjugation site should be carefully chosen in order to obtain optimal immune recognition. As an example, the antigen-MPLA conjugates through 6′-position, where the polysaccharide chain is attached to the natural LPS, were superior in generating IgG responses compared to the antigen-MPLA conjugates that using 1-*O*-position as the conjugation site (Wang et al., 2017), while the blockade of the phosphate group on MPLA completely suppressed the ability for immune activation (Wang et al., 2011). The linkers between the payload and the carbohydrate backbones also played important roles in immune tuning. For example, the oxidative conjugation of mannan and MUC1 FP through imine linkers induced Th1 type immune response and successfully protected mice from tumor growth, while reductive conjugation through amines induced Th2 type immune response without successful tumor protection (Apostolopoulos et al., 1995b). Interestingly, there are examples using the trace amount of mannoproteins (~5% in mannan) for allergen conjugation as allergic vaccines (Manzano et al., 2016; Sirvent et al., 2016). This strategy, taking advantage of other components in polysaccharide mixtures for chemical conjugation, can maintain the intact carbohydrate structure, which may reduce the chance of disturbing the immune activation function. However, the disadvantage of this strategy might be the quality control issue. The protein components may vary batch-to-batch, which may influence the conjugation efficiency, the physical and biological properties of the final materials.

An attractive strategy for future vaccine design can be the combination of different adjuvants that activates the immune system through different receptors. Adjuvants are playing crucial roles in vaccine design, and there have been examples indicating that combining adjuvants with different immune activating mechanisms can trigger additive effects and enhance the vaccine efficacy (Collier et al., 2018; Paßlick et al., 2018). However, cautions need to be taken in combining other adjuvants with the “self-adjuvating” carbohydrates. There are examples indicating the additional adjuvants have negative effects in MPLA and mannan based vaccine conjugates (Wu and Guo, 2006; Wang et al., 2011; Zhou et al., 2014, 2015; Liao et al., 2016; Sirvent et al., 2016; Benito-Villalvilla et al., 2019). Therefore, the external adjuvant needs to be carefully selected. Understanding the detailed mechanism of how multiple adjuvants collaborate with each other can guide future vaccine designs.

## Author Contributions

XH conceived the topic. SL and XH wrote the manuscript.

### Conflict of Interest

The authors declare that the research was conducted in the absence of any commercial or financial relationships that could be construed as a potential conflict of interest.
